# A low-marker density implementation of genomic selection in aquaculture using within-family genomic breeding values

**DOI:** 10.1186/1297-9686-45-39

**Published:** 2013-10-15

**Authors:** Marie Lillehammer, Theo H E Meuwissen, Anna K Sonesson

**Affiliations:** 1 Nofima AS, P.O. Box 210, N-1431, Ås, Norway; 2 Department of Animal and Aquacultural Sciences, University of Life Sciences, P.O. Box 5010, N-1432, Ås, Norway

## Abstract

**Background:**

Genomic selection can increase genetic gain within aquaculture breeding programs, but the high costs related to high-density genotyping of a large number of individuals would make the breeding program expensive. In this study, a low-cost method using low-density genotyping of pre-selected candidates and their sibs was evaluated by stochastic simulation.

**Methods:**

A breeding scheme with selection for two traits, one measured on candidates and one on sibs was simulated. Genomic breeding values were estimated within families and combined with conventional family breeding values for candidates that were pre-selected based on conventional BLUP breeding values. This strategy was compared with a conventional breeding scheme and a full genomic selection program for which genomic breeding values were estimated across the whole population. The effects of marker density, level of pre-selection and number of sibs tested and genotyped for the sib-trait were studied.

**Results:**

Within-family genomic breeding values increased genetic gain by 15% and reduced rate of inbreeding by 15%. Genetic gain was robust to a reduction in marker density, with only moderate reductions, even for very low densities. Pre-selection of candidates down to approximately 10% of the candidates before genotyping also had minor effects on genetic gain, but depended somewhat on marker density. The number of test-individuals, i.e. individuals tested for the sib-trait, affected genetic gain, but the fraction of the test-individuals genotyped only affected the relative contribution of each trait to genetic gain.

**Conclusions:**

A combination of genomic within-family breeding values, based on low-density genotyping, and conventional BLUP family breeding values was shown to be a possible low marker density implementation of genomic selection for species with large full-sib families for which the costs of genotyping must be kept low without compromising the effect of genomic selection on genetic gain.

## Background

A typical breeding goal for salmon contains traits that can be measured directly on the selection candidates, typically growth-related traits, and traits that are measured on sibs of the candidates, typically disease challenge and slaughter traits. Genomic breeding values [1] depend less on own phenotype than traditional BLUP (best linear unbiased prediction) breeding values, and can increase genetic gain, especially for traits that are difficult to improve by conventional selection [2]. Simulations, in which high-density genotyping of a large number of fish was assumed, showed that genomic selection could increase accuracy of selection and genetic gain in aquaculture species through increased selection accuracy, especially for traits measured on sibs of the selection candidates [3]. In aquaculture breeding, the number of selection candidates and test-individuals to genotype is large and thus, full genomic selection is more expensive to implement than in other species, especially regarding the low economic value of each selected individual [4]. Suggested implementations for genomic selection in aquaculture must therefore focus on cost efficiency.

There are three intuitive ways to decrease the genotyping costs in an aquaculture genomic breeding scheme: reduce the number of markers, reduce the number of genotyped selection candidates, and reduce the number of genotyped test-individuals to be used in the reference-population, i.e. animals with phenotypes and genotypes used to estimate marker effects. However, each of these strategies includes a risk of losing the benefit of genomic selection through reduced accuracy of selection, by reducing the number of markers or the size of the reference population [5], or by reducing selection, if too few selection candidates are genotyped to benefit from the genomic breeding values. One way to reduce genotyping costs is to pool DNA from test-individuals with high and low performance, respectively, and thereby analyse two test-samples for each trait, instead of one from each tested individual [6]. The pooling strategy facilitates communal rearing of individuals from different families, but does not contribute to reducing the number of selection candidates to genotype or the number of markers.

Another way to reduce genotyping costs in a family-based breeding program is to estimate genomic breeding values separately within each family [7]. This within-family genomic selection facilitates low-density genotyping, because the required number of markers increases approximately in proportion to effective population size [5]. Hence, within a full-sib group, with an effective population size of 2, genotyping 100 markers should give approximately the same accuracy of the within-family deviation as genotyping 50 000 markers in a population with an effective population size of 1000 [5]. Estimation of a separate within-family component of the breeding value is based on the same principles as imputation [8], but skipping the imputation-step means that no animal needs to be high-density genotyped. It is assumed that family breeding values with high accuracy are available, so that only the within-family component of the breeding value needs to be estimated using genomic information. It is also assumed that only additive genetic effects are taken into account, and that the breeding value of an individual can be estimated as a sum of the family breeding value and the within-family deviation from the family mean. In that case, the sum of conventional family breeding values and within-family genomic breeding values, using the same strategies as in the previously proposed within-family marker-assisted selection [9], could result in a large increase in accuracy with low-density genotyping. The tested strategies to use within-family genomic breeding values are simple extensions of a traditional family-based breeding program that could be implemented without re-structuring the breeding program.

The aim of this study was to estimate the potential of within-family genomic breeding values to increase genetic gain and reduce rate of inbreeding. Alternative strategies considered varying marker densities, levels of pre-selection and number of individuals with phenotypes and genotypes for a trait not measured on the selection candidates. The latter defines the size of the reference population for a trait measured on sibs of the candidates. These strategies were compared to a conventional breeding strategy, using only conventional BLUP breeding values, and a full genomic selection breeding strategy, using only genomic breeding values. Stochastic simulations were used for comparisons and the design resembled an aquaculture breeding population.

## Methods

### Simulation of a historical population

A population with an effective population size of 1000 was simulated for 4000 generations, according to the Fisher-Wright population model to create a historical population in mutation-drift balance. Every individual had a diploid genome with ten 100 cM chromosomes, and polymorphisms were created by random mutations, as in Sonesson and Meuwissen [10], resulting in SNPs randomly distributed over the genome. In the last generation, 100 SNPs from each chromosome (1000 SNPs in total) were chosen randomly from the generated SNPs with a minor allele frequency above 0.05 and assigned to be QTL. We assumed a large number of QTL, each with a small effect, to avoid over-estimation of the information content in the genomic data. Among the remaining SNPs, the 50 or 100 SNPs with the highest minor allele frequencies from each chromosome were assigned as molecular markers to reflect different marker densities. To test marker densities below 50 per Morgan, every second marker, among the 50, was deleted repeatedly to create data with 25, 12, 6 and 3 markers per Morgan. This strategy resulted in low-density marker panels with an adequate distribution over each chromosome.

### Simulation of QTL effects and true breeding values

Two traits were simulated, and each QTL had a simulated effect for these two traits, sampled from a multivariate normal distribution with means 0 and variance $\frac{1}{1000}\left[\begin{array}{cc} \sigma_{\mathrm{gSIB}}^{2} & 0\\ 0 & \sigma_{\mathrm{gCAND}}^{2} \end{array}\right],$ where $\sigma_{\mathrm{gSIB}}^{2}$ and $\sigma_{\mathrm{gCAND}}^{2}$ are the genetic variances (both set to 1) for a trait measured on sibs (SIB) and a trait measured on the selection candidates (CAND), respectively. The true breeding values (TBV_SIB_ and TBV_CAND_) for each individual were calculated as the QTL effects multiplied by the number of copies of a given allele and used to generate phenotypic records. True genetic variances were estimated from the true breeding values, and for all scenarios they were very close to the expectation of 1 for both traits in the first generation before selection started (results not shown). The SIB-records (y_SIB_) were simulated as: y_SIB_ = TBV_SIB_ + e_SIB_, and CAND-records as y_CAND_ = TBV_CAND_ + e_CAND_, where e_CAND_ and e_SIB_ were random numbers sampled from a normal distribution with variances σ^2^_e,CAND_ and σ^2^_e,SIB_, respectively. It was assumed that both traits had a heritability of 0.25, and that the traits were genetically and phenotypically uncorrelated.

### Simulation of the breeding population

The size and breeding structure of the population under selection were based on the salmon breeding population of AquaGen AS (http://aquagen.no), but simplified to facilitate stochastic simulation. A base population of 300 males and 300 females was generated by random mating between individuals from the last generation of the historical population. The base generation animals were mated randomly, creating 300 full-sib families. From each full-sib family, 200 offspring, 100 males and 100 females, were considered as selection candidates and 10, 20 or 33 offspring were considered as test animals, i.e. they obtained phenotypic records for SIB and could not be selected as parents. Subsequent generations were generated by selecting 300 males and 300 females among the selection candidates on their estimated breeding values and performing random mating to form 300 full-sib families in each generation, assuming non-overlapping generations. Equal economic weights were used for the two traits. From each full-sib family, a maximum of 15 females and 15 males were selected, to reduce the rate of inbreeding and avoid large differences in rate of inbreeding between selection strategies.

### Breeding value estimation

Three different kinds of estimated breeding values were used; BLUP-EBV, GS-EBV and COMB-EBV. BLUP-EBV and COMB-EBV were estimated simultaneously for both traits, while GS-EBV was estimated separately for each trait and then combined.

BLUP-EBV were estimated using conventional BLUP methodology, with the model: ***y = Xμ + Zu + e***, where ***y*** was a ((C + S) × 1) vector, where C and S are the total numbers of animals with CAND and SIB records, respectively, accumulating over generations, containing the phenotypes for the two traits CAND and SIB, ***μ*** was a (2 × 1) vector of the overall means for the two traits and ***X*** a ((C + S) × 2) design matrix to assign observations to traits, ***Z*** was a ((C + S) × N) design matrix assigning the observations to N animals, where N is the number of animals in the pedigree, recorded from the first generation of the selection experiment, ***u*** was a (2 N × 1) vector of breeding values, with variance: $\left[\begin{array}{cc} {}_{0}^{\sigma_{\mathrm{gSIB}}^{2}} & {}_{\sigma_{\mathrm{gCAND}}^{2}}^{0} \end{array}\right]⊗A,$ where **A** was the additive relationship matrix between the animals, and **e** was a vector of random residuals with variance $\left[\begin{array}{cc} {}_{0}^{\sigma_{\mathrm{eSIB}}^{2}} & {}_{\sigma_{\mathrm{eCAND}}^{2}}^{0} \end{array}\right]⊗I,$ where **I** is the identity matrix.

GS-EBV were genomic breeding values, estimated separately for each trait using the BLUP-method of Meuwissen et al. [1], also sometimes referred to as genomic blup, which is a two-step approach. First, the marker effects were estimated with the model: $y_{\mathrm{ij}}=\mu_{j}+\sum_{k=1}^{\mathrm{nmrk}}M_{\mathrm{ik}}a_{\mathrm{kj}}+e_{\mathrm{ij}},$ where *y*_*ij*_ was the phenotypic record for individual *i*, trait *j*, *μ*_*j*_ was the mean of trait *j*, *nmrk* was the number of markers, *M*_*ik*_ was the standardised marker genotype for animal i in position *k*, *a*_*kj*_ was the random effect of marker *k* on trait *j*, assumed to have a variance of $\frac{1}{\mathrm{nmrk}}\sigma_{\mathrm{gj}}^{2},$ and *e*_*ij*_ was a random residual for animal *i*, trait *j*. $M_{\mathrm{ik}}=\frac{t_{\mathrm{ik}}-2p_{k}}{\sqrt{2p_{k}\left(1-p_{k}\right)}},$ where *t*_*ik*_ is the number of 1-alleles animal *i* carries, and *p*_*k*_ is the frequency of the 1-allele in the population. Thereafter, GS-EBV were estimated as $\sum_{k=1}^{\mathrm{nmrk}}M_{\mathrm{ik}}{\hat{a}}_{\mathrm{kj}}$ for the selection candidates, where ${\hat{a}}_{\mathrm{kj}}$ is the estimate of the marker effect for marker *k*, trait *j*.

The records used to estimate the marker effects came from all genotyped animals with phenotype, and differed between the two traits. For SIB, the reference population consisted of the genotyped test-individuals, while for CAND, the reference population consisted of genotyped selection candidates with CAND-records. The assumption that the genetic variance is known is not valid in real data, for which additional data might be needed to estimate the genetic variances.

COMB-EBV was a combination of a conventional family estimated breeding value and a genomic within-family breeding value, where the COMB-EBV of individual i was calculated as: ***COMB-EBV***_***il***_ *= ½****a***_***si***_ *+ ½****a***_***di***_ *+* ***w***_***il***_, where ***a***_***si***_ and ***a***_***di***_ were vectors of BLUP-EBV of the sire and dam of individual *i*, respectively, for the two traits, estimated as described in the BLUP-EBV-section, and ***w***_***il***_ was the within-family genomic breeding values for individual *i*, family *l*, for the two traits, estimated for family *l* as ***y***_***l***_ *=* ***X***_***l***_***μ***_***l***_ *+* ***Z***_***l***_***w***_***l***_ *+* ***e***_***l***_, where ***y***_***l***_ was a ((c_l_ + s_l_) × 1) vector of observations from family *l*, where c_l_ was the number of genotyped candidates from family *l* and s_l_ was the number of genotyped test-individuals from family *l*, ***μ***_***l***_ was a vector of means for the two traits for family *l*, ***X***_***l***_ (dimension: (c_l_ + s_l_) × 2) and ***Z***_***l***_ (dimension: (c_l_ + s_l_) × 2(c_l_ + s_l_)) were design matrices, assigning observations from family *l* to traits and individuals within the family, while ***w***_***l***_ was a (2(c_l_ + s_l_) × 1) vector of within-family breeding values from family *l*, expressed as a deviation from the family mean with variance $\frac{1}{2}\left[\begin{array}{cc} {}_{0}^{\sigma_{\mathrm{gSIB}}^{2}} & {}_{\sigma_{\mathrm{gCAND}}^{2}}^{0} \end{array}\right]⊗G_{1},$ where **G**_**l**_ was the genomic relationship matrix for the animals from full-sib family *l*, estimated based on the available markers as ***G***_***l***_ ***= M***_***l***_***M***_***l***_***’/****nmrk*, where ***M***_***l***_ is a matrix of standardised genotypes calculated within family *l*. Other variables were as defined above. When estimating genomic breeding values within a family, only records from that family were used. Therefore, the reference populations for SIB (one for each family) consisted of 33 (in most cases, but fewer in some scenarios described later) test-individuals with records and genotypes, while the reference populations for CAND consisted of the genotyped selection candidates from the actual family and varied in size between families and strategies. None of the estimation methods for genomic breeding values included the own CAND-phenotype of the selection candidate directly, but the information was included in the estimation by including the selection candidate in the reference population for CAND.

### Selection strategies

The within-family genomic selection strategy (W-FAM), a two-step selection scheme designed to take advantage of the family based breeding program and COMB-EBV, which was compared to two different one-step approaches, one that used GS-EBV (GS_POP) and one that used BLUP-EBV (CONV).

The within-family genomic selection strategies (W-FAM) consisted of two steps; pre-selection and final selection. Pre-selection was based on BLUP-EBV after the phenotypes for both traits were obtained. The number of pre-selected males and females, respectively, was varied from 1000 to 30 000 (3% - 100%). Pre-selecting 100% of the selection candidates is equivalent to omitting pre-selection and genotyping all candidates and test-individuals. The pre-selected candidates and all test-individuals from the families in which one or more candidates were pre-selected were genotyped. The pre-selected candidates obtained COMB-EBV, and final selection of 300 male and female parents for the next generation was based on these estimated breeding values. Stronger pre-selection led to fewer families as well as fewer animals within each family selected. The latter had consequences for the reference populations for CAND, which consisted of the genotyped selection candidates. The reference populations for SIB consisted of 33 individuals, irrespective of the level of pre-selection. Scenarios with fewer test-individuals (10 or 20 per family) and the effects of genotyping only a random 50% of these test-individuals were included to study the effects of the number of SIB phenotypes and/or genotypes. Within the W-FAM strategy, marker densities between 3 and 100 markers per Morgan were tested.

For comparison, a conventional strategy (CONV), and a full genomic selection strategy (GS_POP) were used. Both these strategies included only one selection step. In CONV, selection was performed based on BLUP-EBV after phenotypes were obtained for CAND on the candidates and for SIB on the test-individuals, equivalent to the pre-selection step in the W-FAM strategy. In GS_POP, all animals in the population were genotyped, and selection was performed in one step based on GS-EBV. The GS_POP-strategy was tested for two different marker densities, 50 or 100 markers per Morgan. The GS_POP-strategy and the W-FAM strategy with 100% pre-selection were equivalent except for the breeding value estimation method used.

The strategies were evaluated on their accuracy of selection, estimated as the correlation between true and estimated breeding values for the selection candidates, and genetic gain and rate of inbreeding averaged over 10 generations of selection. All results are averages of 50 replicates.

## Results

### Effect of marker density and statistical method

Table 1 summarizes the results for selection accuracy, genetic gain and rate of inbreeding obtained with the different strategies for two marker densities (50 or 100 markers per Morgan) when no (CONV) or all (W-FAM and GS_POP) animals were genotyped. With both marker densities, genotyping all candidates and sibs within the W-FAM strategy for 50 markers per Morgan increased total genetic gain by 15%, compared to CONV, by increasing genetic gain for the SIB and CAND traits by 16% and 14%, respectively. Doubling the marker density to 100 markers per Morgan did not affect the W-FAM-results significantly. Using 50 markers per Morgan, GS_POP gave a reduction in total genetic gain and shifted genetic gain from CAND towards SIB, causing a 16% increase in genetic gain for SIB but a 19% reduction in genetic gain for CAND, compared to CONV. Increasing the marker density to 100 markers per Morgan increased total genetic gain for the GS_POP-strategy by 15%, but W-FAM still outperformed GS_POP when looking at the average over 10 generations. However, it should be noted that GS_POP had an accumulating reference population, while W-FAM re-built the reference populations every generation. GS_POP thereby gave low genetic gains for the first 2-3 generations, but performed similar to W-FAM after a plateau had been reached (results not shown). Even when evaluating the averages over 10 generations, GS_POP obtained a larger increase in genetic gain for SIB than W-FAM, but lower genetic gain for CAND. The difference between these strategies when no pre-selection is applied is how the family component of the breeding value is estimated, using genomics in GS_POP, but conventional BLUP in W-FAM. In both cases, the family component will be more accurately estimated for CAND than for SIB, due to more phenotypic records for CAND within each family and own phenotype for the selection candidates. Conventional BLUP breeding values were more sensitive to this than the genomic breeding values, causing the gap in accuracy between the family components of CAND and SIB to be larger in W-FAM than in GS_POP.

**Table 1:** Results from comparing breeding value estimation methods without pre-selection, for two marker densities

		**50 markers/Morgan**		**100 markers/Morgan**	
	**CONV**^**1**^	**W-FAM**^**2**^	**GS_POP**^**3**^	**W-FAM**^**2**^	**GS_POP**^**3**^
Accuracy of selection	0.56 (0.001)	0.61 (0.001)	0.48 (0.003)	0.62 (0.001)	0.56 (0.003)
Genetic gain (σ_g_) CAND^4^	1.05 (0.011)	1.20 (0.008)	0.85 (0.010)	1.21 (0.010)	0.99 (0.011)
Genetic gain (σ_g_) SIB^5^	0.62 (0.010)	0.72 (0.009)	0.72 (0.013)	0.73 (0.011)	0.83 (0.014)
Total genetic gain (σ_g_)	1.67 (0.011)	1.92 (0.011)	1.58 (0.015)	1.95 (0.013)	1.82 (0.015)
Rate of inbreeding	0.0131 (0.0003)	0.0112 (0.0003)	0.0122 (0.0004)	0.0118 (0.0003)	0.0093 (0.0002)

Standard errors are given in parantheses.

^1^ CONV = conventional breeding strategy without genotyping.

^2^ W-FAM = genomic selection with marker effects estimated within full-sib families and combined with conventional BLUP family breeding values when 100% of the candidates were pre-selected.

^3^ GS_POP = genomic selection with marker effects estimated population wide and no pre-selection.

^4^ A trait measured on the selection candidates themselves.

^5^ A trait measured on full-sibs of the selection candidates.

The effects of breeding strategy and marker density on rate of inbreeding were limited by the restriction on the number of selected parents from each full-sib family. Still, the genomic selection schemes gave a reduction in rate of inbreeding of 7 to 29%, compared to CONV. GS_POP resulted in a higher rate of inbreeding than W-FAM when the marker density was 50 markers per Morgan, but the estimation methods re-ranked at a higher marker density, for which GS_POP gave the lowest rate of inbreeding.

For W-FAM, the effect of marker density was limited, even for a wider range of densities (Table 2). Regardless of trait, marker density had only small effects on the genetic gain down to the density of 12 markers per Morgan. At lower marker densities, genetic gain decreased with decreasing marker density. With only three markers per Morgan, total genetic gain was only 6% greater for W_FAM than for CONV, using less than 50% of the potential of the W-FAM strategy.

**Table 2:** Effect of marker density on accuracy and genetic gain for within-family selection and no pre-selection

**Markers per Morgan**	**Accuracy of selection**	**Genetic gain (σ**_**g**_**)**		
		**CAND**^**1**^	**SIB**^**2**^	**Total**
3	0.58 (0.002)	1.06 (0.010)	0.71 (0.011)	1.77 (0.015)
6	0.59 (0.004)	1.11 (0.010)	0.73 (0.009)	1.84 (0.013)
12	0.60 (0.002)	1.15 (0.009)	0.73 (0.009)	1.88 (0.011)
25	0.61 (0.002)	1.18 (0.012)	0.73 (0.009)	1.91 (0.012)
50	0.61 (0.001)	1.20 (0.008)	0.72 (0.009)	1.92 (0.011)
100	0.62 (0.001)	1.21 (0.010)	0.73 (0.011)	1.95 (0.013)

Selection was based on a sum of conventional BLUP family breeding values and genomic within family breeding values (W-FAM), and all candidates and test-individuals were genotyped. Standard errors are given in parantheses.

^1^ A trait measured on the selection candidates themselves.

^2^ A trait measured on full-sibs of the selection candidates.

### Effect of pre-selection within the W-FAM strategy

When pre-selection was used to reduce the number of genotyped animals, the number of candidates to genotype was fixed while the number of test-individuals to genotype varied with the number of families from which the pre-selected candidates originated. The numbers of genotyped animals per generation are in Table 3 that shows that pre-selection reduced the number of genotyped animals by several thousands. When genotyping 10% of the selection candidates, 94% to 98% of the potential additional gain from the W-FAM strategy was obtained, depending on marker density (Table 4). The effect of pre-selection on total genetic gain increased with increasing marker density, i.e. at low marker densities, a stronger pre-selection could be applied without losing genetic gain. Rate of inbreeding increased by 3 to 29% when 10% of the candidates were pre-selected, compared to genotyping all candidates, and the effect of pre-selection increased as marker density increased up to 50 markers per Morgan (Table 5).

**Table 3:** Average number of animals genotyped per generation for different fractions of pre-selected candidates

**Fraction of pre-selected candidates**	**Number of genotyped animals**
3%	4093
10%	10 206
17%	15 580
20%	18 121
25%	21 804
50%	38 847
100%	70 000

The number of individuals genotyped under the W-FAM strategy when the fraction of pre-selected candidates was varied. The reported numbers are averages over all tested marker densities.

**Table 4:** **Total genetic gain (σ**_**g**_**) for various marker densities and levels of pre-selection for within-family selection**

**Pre-selected**	**CONV**	**Marker density (markers per Morgan)**					
		**3**	**6**	12	25	50	100
3%		1.71 (0.011)	1.74 (0.014)	1.75 (0.014)	1.77 (0.013)	1.78 (0.013)	1.80 (0.015)
10%		1.74 (0.013)	1.81 (0.010)	1.84 (0.013)	1.84 (0.012)	1.85 (0.011)	1.83 (0.011)
17%		1.74 (0.012)	1.82 (0.016)	1.84 (0.011)	1.88 (0.012)	1.89 (0.012)	1.91 (0.012)
20%		1.73 (0.013)	1.82 (0.011)	1.86 (0.012)	1.89 (0.012)	1.89 (0.014)	1.91 (0.011)
25%		1.75 (0.014)	1.83 (0.011)	1.84 (0.011)	1.90 (0.010)	1.89 (0.013)	1.90 (0.011)
50%		1.74 (0.012)	1.84 (0.010)	1.90 (0.013)	1.90 (0.011)	1.92 (0.015)	1.92 (0.015)
100%	1.67 (0.011)	1.77 (0.015)	1.84 (0.013)	1.88 (0.011)	1.91 (0.012)	1.92 (0.011)	1.95 (0.013)

Selection was on a combination of conventional BLUP family breeding values and within family genomic breeding values (W-FAM) at different marker densities. Genetic gain obtained by the conventional breeding strategy (CONV) was included for comparison. Standard errors are given in parentheses.

**Table 5:** Rate of inbreeding (%) with different marker densities and levels of pre-selection for within-family selection

**Pre-selected**	**CONV**	**Marker density (markers per Morgan)**					
		**3**	**6**	**12**	**25**	**50**	**100**
3%		1.34 (0.04)	1.46 (0.04)	1.50 (0.05)	1.45 (0.04)	1.52 (0.04)	1.50 (0.05)
10%		1.36 (0.02)	1.40 (0.03)	1.48 (0.04)	1.42 (0.03)	1.45 (0.03)	1.42 (0.03)
17%		1.39 (0.03)	1.46 (0.04)	1.42 (0.04)	1.36 (0.03)	1.33 (0.03)	1.37 (0.02)
20%		1.36 (0.02)	1.33 (0.03)	1.35 (0.03)	1.37 (0.03)	1.37 (0.03)	1.38 (0.03)
25%		1.33 (0.03)	1.32 (0.03)	1.32 (0.03)	1.34 (0.03)	1.31 (0.03)	1.35 (0.03)
50%		1.34 (0.03)	1.24 (0.03)	1.23 (0.03)	1.17 (0.02)	1.21 (0.03)	1.21 (0.02)
100%	1.31 (0.03)	1.32 (0.03)	1.22 (0.02)	1.24 (0.03)	1.17 (0.02)	1.12 (0.03)	1.18 (0.03)

Selection was on a combination of conventional BLUP family breeding values and within family genomic breeding values (W-FAM) at different marker densities. Rate of inbreeding obtained by the conventional breeding strategy (CONV) was included for comparison. Standard errors are given in parentheses.

### Effect of number of individuals tested for SIB

A method to reduce genotyping costs even more is to reduce the number of sibs tested and/or genotyped from each family. While the number of test-individuals from each family affected total genetic gain, the fraction of test-individuals genotyped affected the relative contribution of each trait to total genetic gain only. Thus, results are shown for SIB only. When a reduced number of test-individuals (10 to 20) was assumed, W-FAM increased genetic gain for SIB by 10 to 13%, compared to CONV, and the difference between genotyping half of or all the test-individuals was small and insignificant (Table 6). When a reference population of 33 was assumed, genetic gain for SIB increased by 26% if all test-individuals were genotyped, but only by 15% if a random 50% of the test-individuals were genotyped, compared to CONV. These results indicate that in a situation for which the potential gain of W-FAM is small, genotyping fewer animals might not reduce the genetic gain, but for a potential larger improvement, more genotyped individuals are needed.

**Table 6:** **Genetic gain (σ**_**g**_**) of SIB**^**1 **^**for varying numbers of test-individuals with phenotypes and/or genotypes for within-family and conventional selection**

**Number of sibs with phenotype**	**W-FAM**^**2**^**– all sibs genotyped**	**W-FAM**^**2**^**– half of sibs genotyped**	**CONV**
10	0.57 (0.012)	0.57 (0.011)	0.52 (0.011)
20	0.68 (0.010)	0.67 (0.011)	0.60 (0.011)
33	0.76 (0.011)	0.71 (0.011)	0.62 (0.010)

Standard errors are given in parentheses.

^1^ A trait measured on full-sibs of the selection candidates

^2^ W-FAM = genomic selection with marker effects estimated within full-sib families and combined with conventional BLUP family breeding values. 10% of the candidates were pre-selected, and pre-selected candidates, and all or half of their test-individuals, were genotyped for 12 markers per Morgan.

## Discussion

The W-FAM strategy uses the typical family-based aquaculture breeding structure with large full-sib families, to obtain accurate conventional breeding values for each family and combine them with genomic within-family breeding values, only using molecular markers to estimate the deviation from the family mean for each candidate. Our hypothesis that W-FAM should require a much lower marker density than GS_POP was confirmed by the results. GS_POP showed a considerable effect of marker density, already between 50 and 100 markers per Morgan, while W-FAM showed no significant effect of marker density on genetic gain until very low marker densities were tested. Both strategies showed an effect of marker density on rate of inbreeding, but the difference in inbreeding between scenarios was limited, because of the common restriction on the number of selected full-sibs. Even at low marker densities, GS_POP and W-FAM schemes gave lower rates of inbreeding than CONV, due to the within-family selection. For W-FAM, the increase in accuracy of selection, compared to CONV, is 9 and 11% for 50 and 100 markers/Morgan, respectively (Table 1), whereas total genetic gain increased by 15 and 17%, respectively. This may be explained by a lower correlation between the estimated breeding values of full-sibs, which increases the selection intensity in two ways: directly [11] and via the constraint on the number of full-sibs selected from each family, which can be seen by the lower rate of inbreeding for W-FAM, compared to CONV. The W-FAM-results are not affected by the effective population size since each full-sib family is treated separately, and the genomic predictions are mainly tracing parents’ chromosome segments rather than population-wide linkage disequilibrium. However, thousands of animals were genotyped for each generation, when summing candidates and test-individuals. For any of the proposed strategies to be cost-effective, the genotyping costs per sample must be quite low, which is facilitated by the use of low-density marker panels.

A pre-selection down to approximately 10% could be performed without losing more than 2 to 6% of the effect of W-FAM on genetic gain. This is in concordance with previously reported results for pigs, where a strong pre-selection before genotyping was recommended for genomic selection when animals had own phenotype before pre-selection was applied [12]. In the present study, phenotypes of candidates were assumed for one of the two traits under selection only, but full-sibs records were available for the other trait at the time of pre-selection and this information was included in the CONV-EBV used for pre-selection. Using W-FAM, pre-selection of candidates caused a reduction in the number of candidates to genotype, as well as test-individuals, because test-individuals from families with no pre-selected candidates were not genotyped. The number of animals genotyped to build a reference population was therefore reduced without reducing the accuracy of selection. However, rate of inbreeding was increased, probably because strong pre-selection increases the amount of between-family selection compared to within-family selection. The relative contribution of the two traits to total genetic gain was little affected by the number of pre-selected candidates (results not shown), an expected result when assuming the traits to be un-correlated and both traits are considered in both selection steps. Tables 4 and 5 together show an interaction between marker density and level of pre-selection, which indicates that marker density is more relevant when more animals are genotyped and that level of pre-selection is also more relevant for higher marker densities.

The number of genotyped sibs from each family affected the relative contribution of each trait to total genetic gain, rather than the total genetic gain. The potential of genomic selection to increase a SIB-trait could therefore be reduced if not all test-individuals are genotyped. Since genotyping more or less test-individuals affected the distribution of genetic gain among traits, the rate of improvement does not depend only on the characteristics of the tested trait but also on the other traits in the breeding goal. The effects of genotyping a fraction of the test-individuals within each family should therefore be tested by including data on all relevant traits before reduced genotyping of test-individuals is applied.

In this study, the strategies were compared under assumptions believed to favour W-FAM over GS_POP, such as large families, a simple family structure and low marker densities. For the first generations, GS_POP also gave low gains due to the lack of a proper reference population until a few generations of reference animals had accumulated. After approximately five generations, GS_POP reached maximum accuracy, and evaluating only generations 5 to 10 caused GS_POP and W-FAM to perform similarly when a marker density of 100 was used (results not shown). For traits that are expensive to phenotype, it might therefore be more cost-effective to perform dense genotyping than to obtain phenotypes for several fish per family, especially in the long run, since GS_POP continuously increases the size of its reference population. For marker densities between 100 to 1000 markers per Morgan, an increase in accuracy of up to 33% was reported when comparing full genomic selection with a conventional strategy [3]. Thus the GS_POP-strategy might also be preferred in cases for which dense genotyping is cheap and available. Between adjacent QTL, r^2^ in the simulated dataset was 0.0353, and r^2^ between a QTL and the closest marker, using 100 markers per Morgan, was 0.0703. The r^2^ between adjacent markers when using 100 markers per Morgan was 0.117, showing a large effect of that we systematically chose SNPs with the highest minor allele frequency as markers. Thus, the required marker density in real data might be higher than indicated by the simulations, because the selection of SNPs on minor allele frequency performed in the simulations causes higher linkage disequilibrium between the SNPs than expected from the SNP density.

The GS_POP-strategy could be performed without separate rearing of families, thereby reducing the bias of estimated breeding values due to common environmental effects and reducing costs related to separate rearing. However, on the one hand, W-FAM requires that the fish have a known pedigree, at least one generation back, because pre-selection is performed prior to genotyping. On the other hand, W-FAM can be implemented within the current breeding scheme of many species which produce large families and collect phenotypes from each family. Implementation can be done at low costs by pre-selection of candidates before genotyping, and by genotyping relevant sibs only at low marker density. Because the required marker density is low, the W-FAM strategy can also be useful for species for which no dense SNP-chips are available. The genomic breeding value estimation in W-FAM can probably be improved by using methods that trace markers back to older ancestors, extending the definition of what is called a family [13]. Also other improvements of the statistical method could increase the performance of W-FAM, such as weighted blending of pedigree and marker information and better use of the own performance information of the candidates.

## Conclusions

The W-FAM strategy was found to be able to increase genetic gain, compared to CONV, both for traits measured on candidates and for traits measured on sibs of the candidates. Pre-selection down to 10% of the candidates and marker densities down to 12 markers per Morgan could be used with only minor reductions in genetic gain. Assuming a family breeding program with large families, within-family genomic selection can be a low-risk and low-cost implementation of genomic selection where the genomic evaluation can be implemented within the current breeding scheme and give additional genetic gain.

## Competing interests

The authors declare that they have no competing interests.

## Authors’ contributions

ML performed the simulations and drafted the manuscript. TM and AK wrote the simulation program. All authors contributed in designing the study, interpreting the results and revising the manuscript and read and approved the final manuscript.
